# Spindle Cell Fibromatosis of the Breast: A Lesson in Malignancy Mimicry

**DOI:** 10.1002/ccr3.73017

**Published:** 2026-06-23

**Authors:** Valerie Fiore, Marc Knezevic‐Maragh, Jovia Williams, Caleb Bonsu, Oluwatito Roberts, Sharon Koehler

**Affiliations:** ^1^ Department of Surgery Episcopal Health Services New York New York USA; ^2^ Department of Medicine Ross University School of Medicine Miramar Florida USA

**Keywords:** β‐catenin, breast fibromatosis, desmoid tumor, spindle cell lesion

## Abstract

Spindle cell fibromatosis of the breast (SCFB) is a rare benign tumor that closely mimics malignancy on imaging and clinical examination. Accurate diagnosis relies on histopathology and immunohistochemistry, particularly β‐catenin staining, to guide appropriate management and avoid overtreatment while addressing its locally aggressive behavior and associated conditions.

## 1 Introduction

Spindle cell fibromatosis of the breast (SCFB), also known as desmoid‐type fibromatosis, is an uncommon, benign tumor that often mimics malignancy due to its infiltrative growth and spiculated appearance on imaging [1]. Although histologically benign, clinical significance lies in its ability to mimic aggressive spindle cell neoplasms and metaplastic carcinomas, making accurate diagnosis essential. Benign considerations include nodular fasciitis, myofibroblastoma, and pseudoangiomatous stromal hyperplasia. Malignant differential diagnoses include metaplastic carcinoma, spindle cell carcinoma, fibrosarcoma, and phyllodes tumor. Recent radiographic and pathological reviews emphasize that spindle cell tumors of the breast often have morphologic features that are difficult to distinguish between benign and malignant entities without considering clinical assessment, imaging results, and histologic findings [2].

These lesions typically occur in women between the ages of 15 and 60, though rare cases have been reported in men [3]. They may arise sporadically or in association with trauma, prior breast surgery, or hormonal influences. Genetic alterations, particularly mutations in APC or CTNNB1, contribute to abnormal β‐catenin signaling and are strongly implicated in the pathogenesis of these conditions [4]. Immunohistochemical markers provide diagnostic clues, particularly in distinguishing fibromatosis from malignancies [5].

Patients usually present with a palpable breast mass, often without systemic symptoms, nipple discharge, or skin changes. On mammography or ultrasound, fibromatosis often presents as an irregular, spiculated, hypoechoic mass and is given a BI‐RADS score of 4 or 5 indicating the need for a tissue biopsy [1]. Radiologic features of fibromatosis may also appear benign but are typically indistinguishable from malignant spindle cell neoplasms.

Histologically, SCFB is characterized by bland spindle cells arranged in fascicles or whorls within a collagenized stroma, with minimal atypia and mitotic activity. The contrast between its bland histology and aggressive, carcinoma‐like growth pattern poses a diagnostic dilemma. The lesion's benign cellular features contrast with its invasive margins and muscle involvement, which can suggest a malignant process to the untrained eye [6].

Diagnosis relies heavily on immunohistochemistry, where nuclear β‐catenin positivity, along with vimentin and smooth muscle actin (SMA) expression, supports fibromatosis [2]. Importantly, the absence of cytokeratin (CK) expression helps distinguish SCFB from key malignant epithelial mimics, particularly fibromatosis‐like metaplastic carcinoma, which may demonstrate deceptively bland spindle‐cell morphology and overlap with benign lesions on limited biopsy [2, 7, 8]. Recent classification systems highlight the importance of incorporating histologic patterns, immunohistochemical markers, and molecular studies into diagnostic workflows [9, 10].

Although histologically benign, breast fibromatosis poses significant management challenges due to its locally aggressive behavior and high recurrence rates. Traditional management has favored wide local excision with negative margins [1]. However, this often results in scarring and undesirable cosmetic outcomes and still carries a risk of recurrence with the potential need for reintervention [1]. Management ranges from observation in stable, asymptomatic patients to surgical excision for symptomatic, enlarging, or invading lesions [1, 3]. Recent algorithms support individualized management strategies based on tumor size, symptoms, and radiologic or pathologic concordance. The following two cases highlight the diagnostic dilemmas, surgical considerations, and follow‐up strategies for SCFB.

## 2 Case Presentation

### 2.1 Case 1

A 42‐year‐old female presented to the emergency department with a 5‐day history of right arm numbness and tingling. She reported a known right breast mass diagnosed 3 months prior, which had been biopsied twice since that time and was being observed by a provider at another facility. She was experiencing shortness of breath and pain with inspiration over the right chest wall. She denied any new changes of the breasts other than occasional tenderness in the region of the mass. She denied fever, weight loss, or any other systemic symptoms. Her surgical history included a benign lower back mass excision two decades earlier. On a physical exam, she had a palpable, immobile, and tender mass in the right breast with no overlying skin or nipple changes.

A recent mammogram revealed scattered bilateral fibroglandular densities and coarse calcifications. In the outer right breast at the nipple plane, there was a 10 × 6 × 6 mm nodular asymmetry. In the posterolateral aspect of the same breast, there was a 13 × 10 × 14 mm nodular asymmetry against the inferior margin of the pectoralis muscle, BI‐RADS 4 (Figure 1). To evaluate the extent, she underwent magnetic resonance imaging (MRI) showing the spiculated mass to be inseparable from and possibly invading the subjacent pectoralis minor musculature (Figure 2).

**FIGURE 1:**
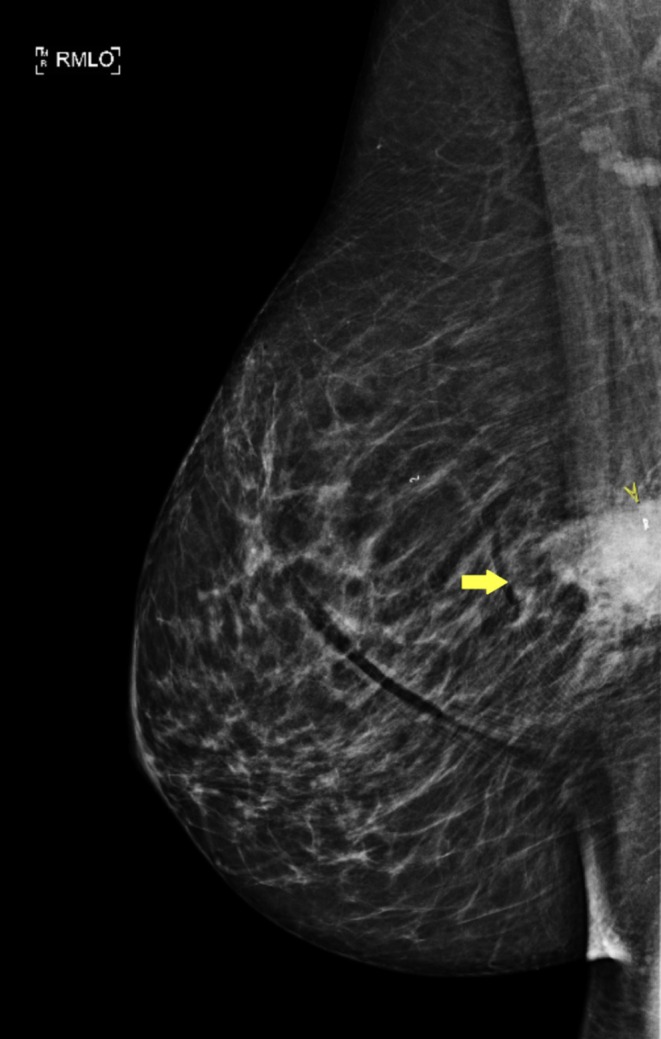
Mammography of the right breast showing a posterolateral 13 × 10 × 14 mm nodular asymmetry against the inferior margin of the pectoralis muscle, BI‐RADS 4 (yellow arrow).

**FIGURE 2:**
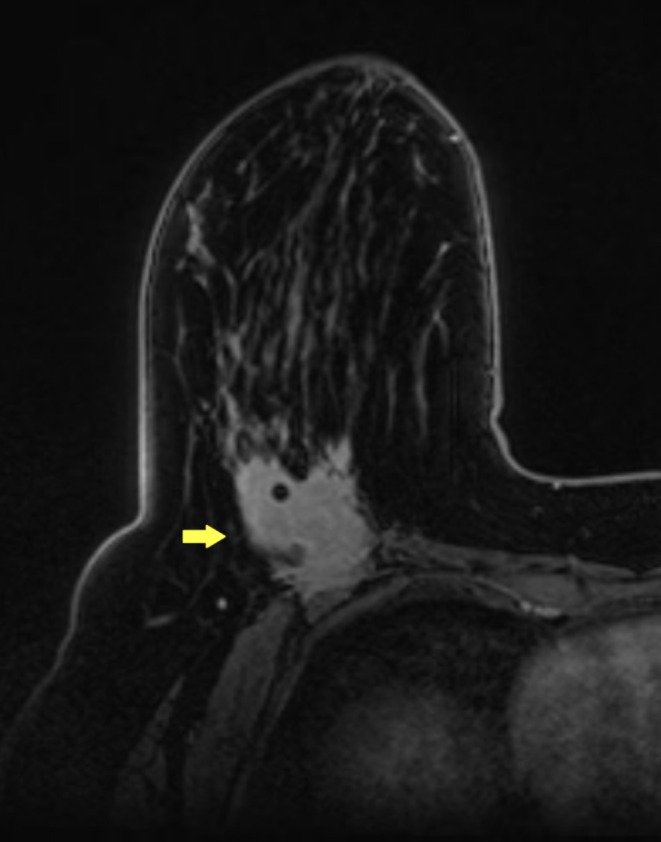
Magnetic resonance imaging (MRI) of the right breast showing a posterolateral spiculated mass abutting and likely invading the adjacent pectoralis musculature (yellow arrow).

Core needle biopsies (CNBs) revealed mild ductal hyperplasia without atypia at the 11:00 position and a fibrotic spindle cell lesion with invasion into the skeletal muscle at the 8:00 position (Figure 3). Immunohistochemical profiling of the 8:00 lesion showed positivity for CK 5/6, estrogen receptor (ER), E‐cadherin, β‐catenin, SMA, and focal actin (Figure 3). A CTNNB1 genomic alteration was detected, as well as low tumor mutation burden and negative microsatellite instability (MSI).

**FIGURE 3:**
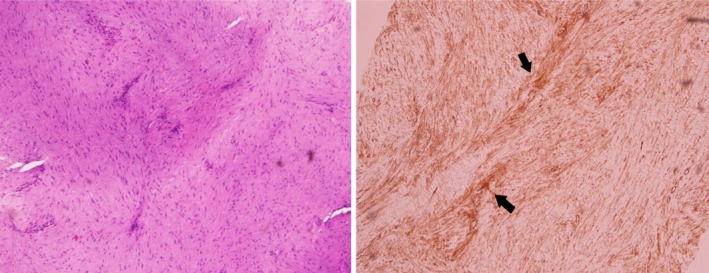
Left: Hematoxylin and eosin (H&E) stain showing interlacing fascicles of bland spindle cells with eosinophilic cytoplasm and elongated nuclei, consistent with a benign spindle cell lesion. Right: Immunohistochemical staining demonstrating nuclear β‐catenin positivity (black arrows).

Due to the invasive nature of the lesion, the patient was referred to a facility capable of thoracic reconstruction, where she subsequently underwent wide surgical excision of the mass. Intraoperatively, the lesion was noted to involve the scalene and pectoralis muscles, which were partially sacrificed in the dissection. The ribs and intercostal muscles were spared. Excision of a 6 × 5.5 × 2.7 cm lesion was achieved with a 2 cm margin, and clips were placed at the margin edges in the event a re‐excision was required.

Final pathology demonstrated desmoid‐type fibromatosis involving the breast parenchyma and skeletal muscle, with tumor present at the deep surgical margin, indicating incomplete resection. Histopathologic evaluation revealed spindle cell proliferation with myofibroblastic differentiation and nuclear β‐catenin expression, consistent with SCFB. Molecular testing further supported the diagnosis, demonstrating a CTNNB1 mutation, while sequencing was negative for MSI, MET, and PTEN alterations.

Postoperatively, the case was complicated by seroma with benign fluid on aspiration. The patient required multidisciplinary management, including surgical follow‐up, referral to oncology, and to gastroenterology for a colonoscopy. Oncology recommended observation and consideration of radiation therapy if there is recurrence and genetic counseling for associated syndromes. She underwent MRI of the chest to evaluate the seroma postoperatively, which demonstrated a decrease in its size, without any areas of suspicious enhancement or lymphadenopathy bilaterally. On postoperative staging computerized tomography (CT), the patient was noted to have no metastatic disease; however, a large intrauterine mass and adnexal lesions were seen. She underwent laparoscopic hysterectomy with bilateral salpingo‐oophorectomy, which revealed a fibroid uterus and benign ovarian lesions. She continues to demonstrate no SCFB recurrence on annual mammography.

### 2.2 Case 2

A 30‐year‐old male with a past medical history of asthma presented with an incidental right breast mass during an emergency department visit for unrelated leg pain. The patient denied breast pain, nipple discharge, physical trauma to his pectoral area, or systemic symptoms. His family history was significant for breast cancer in two maternal aunts, one of whom passed away from breast carcinoma. On a physical exam, a firm, tender, immobile mass was palpated in the right outer lower quadrant with no visible skin changes, nipple retraction, discharge, or supraclavicular lymphadenopathy.

A bilateral diagnostic mammogram revealed a 3.9 × 2.7 × 4.9 cm hypoechoic mass at the 7:00 to 8:00 position of the right breast, 6 cm from the nipple with lobulated margins and no internal vascularity, BI‐RADS 4. An ultrasound‐guided CNB was completed, revealing SCFB (Figure 4). Given the lesion's location near the chest wall, further evaluation with a contrast‐enhanced chest CT was performed, revealing a 3.6 × 3.1 × 3.1 cm soft tissue mass along the right chest wall at the level of the fifth to sixth ribs, extending anteriorly to the subcutaneous tissue but separated from the pectoralis muscle.

**FIGURE 4:**
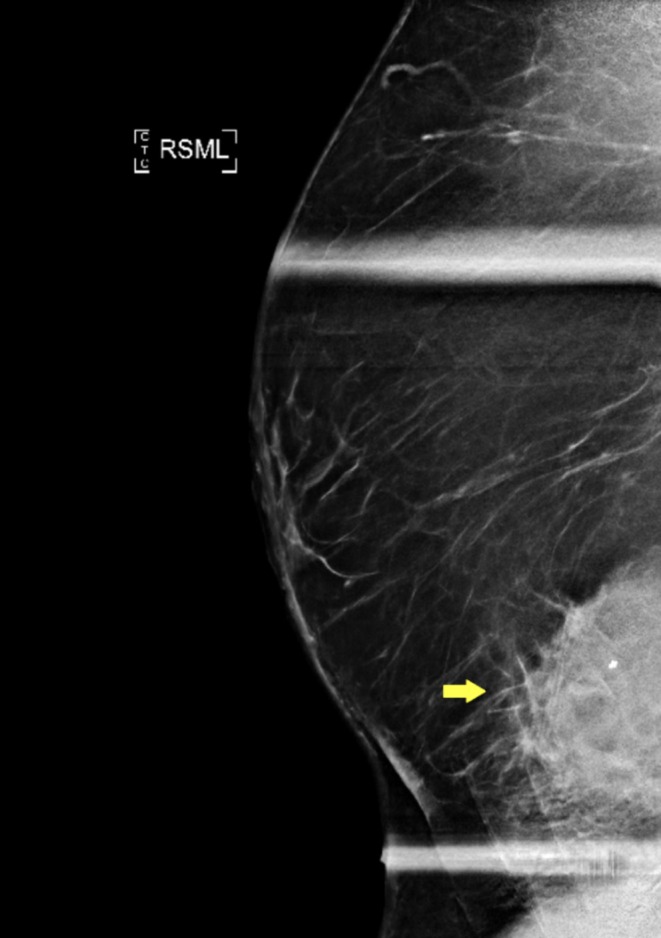
Mammography status post successful ultrasound‐guided core biopsy of the right breast mass, with a barbell clip placement (yellow arrow).

The patient underwent excisional biopsy of the mass and specimen radiography demonstrated a soft tissue mass measuring approximately 5 × 4 × 3.5 cm with negative margins (Figure 5). Histologic evaluation revealed benign spindle‐cell proliferation composed of fibroblasts and myofibroblasts arranged in sweeping fascicles within a collagenous stroma, consistent with spindle cell fibromatosis (Figure 6). Immunohistochemical staining supported the diagnosis, demonstrating positivity for β‐catenin, SMA, vimentin, and cyclin D1, and negativity for CK AE1/AE3, S‐100, and desmin, with a low Ki‐67 proliferation index (Figure 6).

**FIGURE 5:**
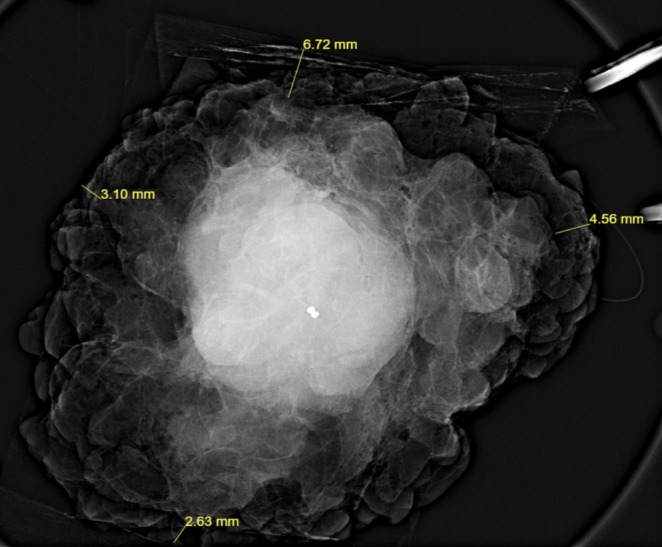
Successful surgical excision of a biopsy‐proven mass measuring approximately 5 × 4 × 3.5 cm with negative margins.

**FIGURE 6:**
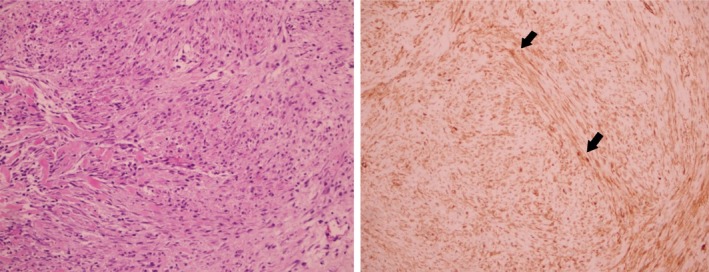
Left: H&E stain demonstrating long, sweeping fascicles of bland spindle cells with elongated nuclei and minimal atypia within a collagenous stroma, consistent with fibromatosis. Right: Immunohistochemical staining demonstrating nuclear β‐catenin positivity (black arrows).

One month following surgical management, the patient developed progressive abdominal pain, prompting further evaluation. Cross‐sectional imaging demonstrated multiple mesenteric and intra‐abdominal masses, as well as a persistent chest wall lesion, concerning for recurrent desmoid disease. Given the constellation of findings, the patient underwent gastrointestinal workup and was found to have diffuse polyposis on upper and lower endoscopy, with genetic testing confirming a pathogenic APC mutation consistent with familial adenomatous polyposis (FAP). Given the presence of extensive polyposis, the patient was counseled on the high lifetime risk of colorectal cancer and the potential need for prophylactic colectomy, as well as consideration of systemic therapy. The patient continues to follow up with a multidisciplinary team at a cancer center.

## 3 Discussion

SCFB can mimic malignancy, both radiologically and pathologically. Irregular and spiculated borders, along with an infiltrative growth pattern, typically warrant tissue sampling [1, 2]. Low to no vascularity on color doppler sonography may give a clue to the benign nature of the tumor; however, it may not always be a reliable criterion for diagnosis. Histologically, SCFB is characterized by bland‐appearing spindle cells with low cellularity, yet it may grow in an infiltrative manner into adjacent soft tissues, including skeletal muscle. A low mitotic rate helps differentiate fibromatosis from metaplastic carcinoma or fibrosarcoma [5]. Evidently, morphological overlap between benign and malignant spindle cell lesions remains a challenging issue.

Fibromatosis‐like metaplastic carcinoma is a malignant entity that closely mimics SCFB, as both may demonstrate the aforementioned histological attributes, but this carcinoma is characterized by CK expression, as well as negative β‐catenin and hormonal expression. This differential is worth noting because it can pose difficulties in diagnosis and management [7, 10]. Therefore, careful histopathological evaluation using a broad immunohistochemical panel, as well as correlation with clinicoradiological findings, is mandatory for an accurate diagnosis. In the two cases described, the radiological features were suggestive of malignancy; however, CNB showed a spindle cell lesion suggestive of SCFB.

The clinical manifestations of the two cases were quite different. Case 1 had locally advanced disease with invasion into adjacent structures and presented with neurologic and respiratory symptoms, despite having been asymptomatic and biopsied 3 months prior with a plan to observe. Case 2 had an incidentally detected localized mass without signs of invasion or symptoms of mass effect, but quickly recurred despite resection with negative margins. This demonstrates how rapid SCFB can progress and how persistent it may be following successful resection [11]. Case 2 further highlights the importance of evaluating systemic or syndromic disease in patients with fibromatosis, such as FAP. In such cases, management extends beyond local tumor control and requires multidisciplinary coordination, including genetic evaluation, gastrointestinal surveillance, and consideration of systemic therapies. Emerging therapies, including gamma‐secretase inhibitors such as nirogacestat, have shown promise in managing unresectable or progressive desmoid tumors in this setting [12]. Recognition of this association is critical, as desmoid tumors in patients with concomitant FAP have demonstrated more aggressive or recurrent disease [13].

In Case 1, positive ER staining is noteworthy. The staining was limited in extent and intensity, and its positivity in fibromatosis should be interpreted with caution. ER positivity may reflect stromal or entrapped epithelial elements rather than true tumor expression; however, ERβ expression has been found to be correlated with higher rates of proliferation in SCFB [14, 15]. Similar findings were drawn in fibromatosis with cyclin D1 immunopositivity [14]. Furthermore, positivity for CK 5/6 may be misleading on CNB because this marker can be expressed in myoepithelial cells and in spindle cell carcinomas, including metaplastic carcinoma [1, 7, 16, 17]. Therefore, the use of a panel of immunohistochemical markers is helpful for the accurate diagnosis of SCFB.

Although wide local excision with negative margins has been considered the treatment of choice for SCFB, observation may be indicated in asymptomatic patients with stable disease [1]. Margin status is important; however, desmoid tumors have a significant rate of recurrence despite achieving negative margins, and clear predictive factors for this have not been delineated [11]. This further places an emphasis on the effectiveness of longitudinal follow‐up in this patient population. Management decisions should incorporate symptom burden, extent of disease, and in patients who have had recurrence, margin status after resection, rather than relying solely on histologic benignity. Current approaches include serial imaging every 3–6 months for the first 2 years and then continuing surveillance with annual MRI [1]. Overall, the discussed cases emphasize that follow‐up strategies benefit from an individualized approach.

## 4 Conclusion

These cases underscore the importance of an interdisciplinary approach to SCFB, particularly in community hospital settings where coordination of diagnostic and surgical care is crucial. Awareness and recognition of SCFB and its mimics are necessary to avoid misdiagnosis and overly aggressive treatment. Immunohistochemistry remains central to diagnosis. Management is tailored to symptom relief, with wide local excision and negative margins as the commonly‐practiced standard for symptomatic or invasive lesions. Interval surveillance is an option for stable cases. Future studies are needed to refine recurrence‐based surveillance algorithms and to clarify the role of adjuvant therapies in select patient subgroups.

Ethics and Patient Consent: This case series was conducted in accordance with the principles of the Declaration of Helsinki of 1964 and its later amendments. Written informed consent was obtained from both patients for publication of this case report and accompanying images. Identifying information has been omitted to ensure patient anonymity. Institutional ethical approval was not required for this publication.

## Author Contributions

**Valerie Fiore:** investigation, methodology, project administration, resources, supervision, writing – review and editing. **Marc Knezevic‐Maragh:** data curation, formal analysis, investigation, writing – original draft, writing – review and editing. **Jovia Williams:** formal analysis, writing – review and editing. **Caleb Bonsu:** formal analysis, writing – review and editing. **Oluwatito Roberts:** formal analysis, writing – review and editing. **Sharon Koehler:** supervision.

## Funding

The authors have nothing to report.

## Conflicts of Interest

The authors declare no conflicts of interest.

## Data Availability

The data that support the findings of this study are derived from the clinical records of the reported patients. Due to the nature of this research and to protect patient privacy, these data are not publicly available. All relevant information is included within the article.
